# Effects of Dexmedetomidine on Basic Cardiac Electrophysiology in Adults; a Descriptive Review and a Prospective Case Study

**DOI:** 10.3390/ph15111372

**Published:** 2022-11-08

**Authors:** Reino Pöyhiä, Teija Nieminen, Ville W. T. Tuompo, Hannu Parikka

**Affiliations:** 1 Palliative Medicine, Department of Clinical Medicine, Kuopio Campus, University of Eastern Finland, 70211 Kuopio, Finland; 2 Department of Anaesthesia and Intensive Care Medicine, Helsinki University Central Hospital, 00280 Helsinki, Finland; 3 Palliative Center, Essote, The South Savo Social and Health Care Authority, 50100 Mikkeli, Finland; 4 University of Oulu, 90570 Oulu, Finland; 5 Department of Cardiology, Helsinki University Central Hospital, 00280 Helsinki, Finland

**Keywords:** dexmedetomidine, fibrillation, ablation, sedation, electrophysiologic, organ protection

## Abstract

Dexmedetomidine (DEX) is a commonly used sedative agent with no or minimal effects on breathing. DEX may also be beneficial in myocardial protection. Since the mechanisms of cardiac effects are not well known, we carried out a descriptive review and examined the effects of DEX on myocardial electrical conduction in a prospective and controlled manner. For the review, clinical studies exploring DEX in myocardial protection published between 2020-2022 were explored. A case study included 11 consecutive patients at a median (range) age of 48 (38–59), scheduled for elective radiofrequency ablation of paroxysmal atrial fibrillation. A bolus dose of DEX 1 µg/kg given in 15 min was followed by a continuous infusion of 0.2–0.7 µg/kg/h. Direct intracardiac electrophysiologic measurements, hemodynamics and oxygenation were measured before and after the DEX bolus. Experimental studies show that DEX protects the heart both via stabilizing cardiac electrophysiology and reducing apoptosis and autophagy after cell injury. The clinical evidence shows that DEX provides cardiac protection during different surgeries. In a clinical study, DEX increased the corrected sinus node recovery time, prolongated the atrioventricular (AV) nodal refractory period and cycle length producing AV nodal Wenckebach retrograde conduction block. DEX has a putative role in organ protection against hypoxic, oxidative and reperfusion injury. DEX slows down the firing of the sinus node and prolongs AV refractoriness.

## 1. Introduction

Dexmedetomidine (DEX) is a selective α2-adrenergic receptor (α2-AR) agonist with a high affinity on the 2A and 2C subtypes of the receptor [1]. DEX produces analgesia and sedation, reduces delirium and agitation, provides stabilizing effects on cardiovascular functions, and causes perioperative sympatholysis. DEX has been widely used for algo-sedation in various invasive surgical, radiological and cardiac procedures, due to easy arousability from sedation and minimal or no effects on spontaneous breathing [2,3,4]. During general anesthesia, DEX reduces the need of volatile and intravenous (iv) anesthetics [4]. The use of DEX is currently studied in palliative care, psychiatry and prevention of arrythmias [5,6,7]. Perhaps one of the most interesting targets of DEX would be prevention of multiorgan failure [7] occurring during surgery, trauma or in intensive care. DEX has a wide therapeutic dose window and can be administered intravenously, subcutaneously, intranasally and sublingually in doses [8]. Spinal and sublingual administration of DEX are currently examined [9,10]. The common dosage of DEX has been 0.25–1 mcg/kg iv, followed by an infusion of 0.2–1.4 mcg/kg/h titrated to the intended effect [11,12,13]. Bolus doses for other routes than iv vary largely and depend on the desired effect.

Bradycardia and hypotension are the most common side-effects that may restrict the use of DEX (Table 1), although in higher doses they may be less pronounced [1,2,3,12,14,15,16,17,18,19,20,21]. That is why in a recent review of sedation techniques for radiofrequency (RF) ablation of aberrant conduits in paroxysmal atrial fibrillation the use of DEX was recommended with caution [22]. Even asystole during DEX administration has been reported [23]. 

Both central and intracardiac mechanisms have been proposed for the possible influences of DEX on the heart. Firstly, bradycardia could be caused by reflectory vasoconstriction due to reduced noradrenaline secretion following the activation of α2A-receptors in locus coeruleus [24]. Presynaptic activation of central α2-receptors results in a sympatholytic effect leading to hypotension and bradycardia, while postsynaptic activation in the peripheral vasculature leads to vasoconstriction and hypertension [24,25,26]. Secondly, DEX has been shown to decrease the intracellular levels of cAMP via G-protein activation, which reduces the activity in the ion channels of cardiac myocytes [27]. An important mechanism of vasodilatation after DEX administration is the activation of endothelial nitric oxide synthase [28]. An α2-receptor mediated baroreceptor reflex and enhanced vagal activity may also count for DEX-induced bradycardia [25,28,29]. In a famous study, Kamibayashi et al. [25] demonstrated that DEX had no influence on the heart rate in vagotomized dogs.

Yet the mechanisms of DEX-induced arrythmias remain to be exclusively examined. The same intracellular mechanisms may be activated in DEX-induced balancing of cardiac electrophysiology and broad myocardial protection [7]. To analyze the most recent evidence of the cardiovascular effects of DEX, we performed a descriptive review. Because only few studies have been executed on the effects of DEX on the myocardial conductivity in adults [30,31], we also aimed to examine the electrophysiologic cardiac effect of DEX during elective ablation of paroxysmal atrial fibrillation.

## 2. Results

### 2.1. Descriptive Review of the Effects of DEX on Cardiac Functions

#### 2.1.1. Experimental Evidence on New Concepts

**Figure 1:**
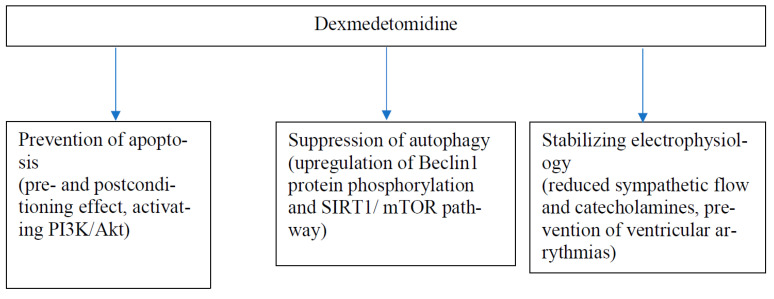
A model for main mechanisms (with two examples) of cardiac protection of DEX.

In vitro studies with isolated rabbit and rat hearts have shown that DEX inhibits voltage-gated Na channels (Nav1.5) and L-type calcium channels (I_C-L_) and opens large-conductance Ca^2+^-sensitive potassium (BKCa) channels, which are important mediators in myocardial conduction [32,33,34]. In addition, DEX has been shown to inhibit pacemaker cells in sinoatrial nodes and reduce ventricular arrythmias [35,36,37]. These effects were only partly or not all reversed by the α-2-adrenergic antagonist, yohimbine, suggesting a direct effect of DEX on the heart. α2-receptors are important in the development of myocardial hypertrophy but nodal tissue lacks α2-receptors [38,39]. In addition, Xia et al. have shown that DEX reduced bupivacaine-induced arrythmias and increased tolerance to bupivacaine-induced cardiac toxicity in rats [40]. There is a growing bulk of evidence supporting the cardiac and other organ protective effects of DEX as proven by several experimental models of ischemic injury and via multiple mechanisms [7]. Examples of the most recent findings [41,42,43,44,45,46,47,48,49,50,51] in the mechanisms of DEX-induced cardioprotection are given in Table 2. DEX may be beneficial for a failing heart given both before (preconditioning-like effect) and after (postconditioning-like effect) the ischemic injury. However, the dose–response relationship of DEX was not clear in the studies. The results of this study propose that the cumulated evidence on the cardiac protection caused by DEX at the cellular level could be divided into the following main categories: inhibition of apoptosis, reversion of autophagy, and stabilization of cardiac electrophysiology after cellular injury (Figure 1) [7,52].

#### 2.1.2. Clinical Significance

The loading dose of dexmedetomidine results in a transient increase in blood pressure and a reflexive drop in heart rate, especially in young, healthy individuals [53]. This initial cardiovascular response is most likely due to vasoconstriction induced by the stimulation of peripheral α-2B receptors in vascular smooth muscle; however, subsequent hypotension occurs when the vasodilatory effects of the central α-2A receptors predominate. 

Due to its sympatholytic properties and ability to activate the parasympathetic tone, DEX might reduce arrythmias during and after surgery and in the intensive care. Recently, three meta-analyses and systematic reviews have been published with different results on the effects of DEX on the prevention of atrial fibrillation [18,54,55]. While two reviews found no significant effect of DEX on arrythmias after cardiac surgery, Wang et al. reported a 17% reduction in AF, 70% reduction in supraventricular tachycardia and an 80% reduction in ventricular arrythmias by DEX in the intensive care units. Another recent review [16] found that DEX effectively reduces ventricular arrythmias, but was based only on six studies with a total number of 1001 patients.

DEX seems to stabilize cardiovascular functions and protect the heart from reperfusion injury in humans. The most recent systematic review by Chen et al. [2022], based on 9 controlled studies including a total of 418 patients, found out that DEX reduced CK-MB, IL6 and TNF-α levels up to 24 h after open-heart surgery performed with CPB. Additionally, it shortened the length of patients’ intensive care department stays, but it had no influence on the total length of the stay at the hospital [56]. The original studies had a very low bias but the dosing regimen of DEX varied greatly. Yet, in all but one trial the administration started in the induction phase and lasted at least until the end of the procedure. Another review, examining the use of DEX during hysterectomies, explored 22 studies including a total of 857 women and concluded that DEX was associated with more stable haemodynamics observed as less drastic fluctuations in blood pressure and heart rate during surgery [57]. This review also demonstrated a very low bias risk for the included studies. Rather similar results were obtained in a prospective, randomized and placebo-controlled study assessing the effects of a bolus dose of 0.6 μg/kg of DEX iv in the induction of anesthesia for laparoscopic cholecystectomy [58]. In this study, the patients receiving DEX experienced also less cough during emergence and less postoperative pain than the patients treated with placebo.

### 2.2. Clinical Study

Our clinical study included two females and nine males at a median (range) age of 48 (38–59) years. Their median weights and heights (with ranges) were 88 (60–108) kgs and 183 (161–196) cm, respectively. All patients had SR during the measurements. During cannulation the patients received a median dose of 1 (range 0.5–2) mg of alfentanil and a median volume of 707 (range 606–716) mL of fluids. The haemodynamic and oxygenation measurements are given in Table 3. The electrophysiological measurements are given in Table 4. No ventricular arrythmias were detected during the procedures.

## 3. Discussion

The main finding of our clinical case study is that infusion of DEX depressed both sinus and AV nodal functions in patients undergoing atrial fibrillation ablation with no bradycardia or hypotension. A significant prolongation in the corrected sinus node recovery time and the AV nodal antegrade refractoriness were observed after a DEX infusion in a situation where haemodynamic fluctuations were eliminated. 

Our findings agree with two other cardiac catheterization studies in adults, although the measurements in those studies were performed only after DEX administration [30,31]. A prolongation of the AV nodal effective refractory period, the AV node block cycle length and the AH interval but not of the QTc time were also observed in all these studies. The physiological role of the AV node is to slow down the impulse propagation between atrial and ventricular tissues. This decremental function was achieved under strict autonomous nervous control. An increase in AV nodal refractoriness, as shown in our study, further restricts any conduction through the node by cutting down the number of atrial impulses entering the node. During sinus rhythm, DEX might lead to an AV block but in atrial tachyarrhythmias, such as atrial fibrillation, it can reduce the ventricular rate and thus, be protective. [59]. A longer ventricular refractory period may be beneficial since the opposite has been associated with an increased risk of ventricular extrasystolia [60]. In 2007, Fox and colleagues calculated that a 10-bpm reduction in HR produces a 30% reduction in the relative risk of cardiac death after a heart infarction [61]. Yet, sinus bradycardia is recognized as a risk factor for AF and ventricular dysrhythmias, particularly if the refractory period is shortened or the QT time is prolonged [62,63]. A pathological sinus bradycardia is a serious cause of morbidity and mortality in situations such as the sick sinus, Brugada or Wollf-Parkinson-White syndromes [64,65]. Neither bradycardia nor prolongation of the QT time were observed in the current study. Significant hypotension due to sympatholysis was not observed in the current study because of standardized prophylactic hydration in our patients. 

The representativeness of the current study is limited by the small number of patients and the lack of placebo. However, our study is the first of its kind examining the electrical conductivity before and after the administration of DEX in adults. Prior to any electrophysiological measurements, our patients received only paracetamol and diazepine orally but no other sedative or analgesic medications. Thus, instead of a parallel placebo-group our patients served as their own controls. In addition, we used a typical loading dose and infusion of DEX, administered only after the baseline measurements had been performed. No ventricular arrythmias during procedures were observed. The clinical implications of the electrophysiological findings on arrhythmia incidence in our study must be drawn cautiously since specific programmed arrhythmia stimulation protocols were not utilized. Although we did not observe significant bradycardia and hypotension, these side-effects occur commonly in the clinical use of DEX (Table 3). However, reduced heart rate has been shown to diminish the occurrence of myocardial injury after an experimental ischaemia and the risk of ventricular arrythmias, probably due to decreased metabolism in cardiomyocytes [66,67,68]. 

## 4. Materials and Methods

### 4.1. Descriptive Review

For the descriptive mini-review, PubMed, Cochrane, and EMBASE were searched for meta-analyses, critical and systematic reviews on the clinical effects of DEX on cardiac functions published between 2020 and 2022. The following search terms were applied: dexmedetomidine, α2-receptors and agonists, cardiology, cardiac protection, arrythmia, myocardial injury. The search was extended to include the most recent experimental evidence on DEX-induced protective myocardial effects. Only peer-reviewed articles in English were accepted. 

### 4.2. Prospective Clinical Case Study

The clinical study was performed following the principles defined in the Helsinki Declaration, and it was registered in the European Union Drug Regulating Authorities Clinical Trials Database, Eudract, with a register number EudraCT 2010-020782-24. After the approval of the institutional Ethics Committee (25 August 2010, 137/13/03/01/2010), a written informed consent was obtained from 11 consecutive patients scheduled for elective RF energy catheter ablation of pAF in a tertiary hospital. Patients with a suspected opioid or benzodiazepine addiction or allergy, on amiodarone, having AF during the procedure, aged over 70, with a known allergy to DEX or a tendency to hypotension were excluded. Antiarrhythmic drugs were discontinued at least five half-lives prior to the clinical study.

One hour before the procedure all patients received 1 g paracetamol and 10 mg diazepam orally as premedication. Femoral cannulation was performed under local anesthesia (lidocaine 1%). If needed, alfentanil was used as the rescue analgesic in 0.25–0.50 mg bolus doses during the cannulation, but not when the electrophysiological measurements were performed. The procedural sedation was performed using a bolus DEX dose of 1 µg/kg given in 15 min, followed by a continuous DEX infusion of 0.2–0.7 µg/kg/h. 

During patient preparation, a Ringer-Acetat (RAC)-infusion of 20 mL/h was introduced and continued until the end of the procedure. To prevent hypovolemia, 6% Hydroxy-Ethyl starch (HES) 500 mL was infused in one hour. If the patient became hypotonic, a more rapid infusion of RAC could be given. In addition, during the procedure, patients were given a Glucose–Electrolyte solution (G5% Na0.3) infusion of 30 mL/h for the femoral vein socket clearing, an RAC infusion of 120 mL/h for the transseptal sheet flushing and an RAC infusion of 2–10 mL/h for the magnetic catheter flushing.

#### Electrophysiology

The electrophysiological measurements were performed in the fasting state. Two quadripolar electrode catheters (Supreme™ CRD, St. Jude Medical Company, St. Paul, MN, USA) were introduced via femoral access to the right ventricular apex and His bundle region, respectively. A deflectable decapolar catheter (Inquiry™, Irvine Biomedical, Inc., St. Jude Medical Company, Irvine, CA, USA) was inserted into the coronary sinus (CS). A bipolar pacing from the most proximal CS pair of electrodes was used for atrial pacing and the ventricular and VA conduction studies were accomplished by RV apical pacing. Care was used to record stable His bundle electrograms. Incremental pacing and single extrastimulus techniques were used to determine the antegrade and retrograde AV conduction properties and the atrial and ventricular effective refractory periods, as well as sinus node recovery time (SNRT) and corrected sinus node recovery time (CSNRT). Pacing was performed with a programmable stimulator (EP-4™ Cardiac Stimulator (St. Jude medical, Inc., St. Paul, MN, USA) using stimuli of 2 ms duration and amplitudes twice the diastolic threshold, which was defined also after the dexmedetomidine infusion. The programmed electrical stimulation was performed prior to transseptal puncture, in the baseline and in the steady state on continuous dexmedetomidine infusion. The protocol and definitions used for the stimulation program were as utilized in clinical studies [69]. In short, an incremental atrial and right ventricular extrastimulus technique with driving cycle lengths of 600 ms and 400 ms was used to determine the atrial and ventricular effective refractory periods (AERP and VERP, respectively), as well as the AV nodal antegrade and retrograde effective refractory periods (AVN ERP600, AVNERP400 and AVNERPret, respectively). Incremental atrial and right ventricular pacing were used to define the cycle lengths producing AV nodal Wenckebach antegrade (AVNW) and retrograde (AVNWret) conduction blocks, respectively. Conduction over the accessory pathway or dual AV conduction physiology were not found in either direction. To determine the effect of dexmedetomidine on the sinus node function, overdrive suppression with rapid atrial pacing at a cycle length of 100 ms shorter than the spontaneous sinus rate for one minute was used. From the return cycle length sinus node recovery time (SNRT) and corrected SNRT (CSNRT) were calculated. 

A 12 lead ECG was recorded during the stable sinus rhythm just prior to the baseline electrophysiological study, and repeated after the completion of the clinical study drug infusion and before starting the second set of electrophysiological measurements. Systolic arterial pressure (SAP), diastolic arterial pressure (DAP), mean arterial pressure (MAP) and heart rate (HR) were measured non-invasively. Peripheral arterial blood saturation (SpO2) was measured with pulse-oximetry.

All measurements were performed twice, before and after the DEX bolus. The data on stimulations and recordings were stored digitally and analyzed off-line, using the mean values of five consecutive measurements. The Wilcoxon signed-rank test was used for pairwise comparisons of statistically significant (*p* < 0.05) differences of hemodynamic, oxygenation and electrophysiological measurements.

## 5. Conclusions

The most recent experimental studies suggest both an α2-receptor related and a direct effect on cardiomyocyte mechanisms for DEX-induced cardiac protection for oxidative and hypoxic injuries of the heart. In surgical and intensive care, DEX has been shown to reduce the incidence of ventricular arrythmias and to stabilize haemodynamics in humans. Further studies are needed to establish the role of DEX in clinical organ protection both after surgery and myocardial infarction. Our clinical study shows that DEX has a slight inhibitory effect on sinus node function, and that it prolongs atrioventricular refractoriness in patients undergoing atrial fibrillation ablation. Whether these are direct actions remains unclear, but if proper hydration is provided and hypotension avoided, the response is evident.

## Figures and Tables

**Table 1:** Risk of bradycardia and hypotension in recent meta-analyses and systematic reviews. N = number of patients, RR = relative risks, ICU = intensive care unit, LCC = laparoscopic cholecystectomy, NIV = non-invasive ventilation, PACU = postanesthesia care unit.

Study	Settings	N of Patients/Trials	RR for Hypotension (95% CI)	RR for Bradycardia (95% CI)
Miao et al., 2020 [12]	non-cardiac surgery	682/9	1.48 (0.68–3.23)	1.78 (0.78–4.02)
Bi et al., 2021 [14]	non-cardiac surgery, elderly patients	4376/16	1.29 (1.12–1.49)	1.39 (1.15–1.67)
Lewis et al., 2021 [15]	sedation for NIV in ICU	738/12	1.98 (1.32–2.98)	2.80 (1.92–4.07)
Zhong et al., 2022 [16]	cardiac surgery	1001/7	1.10 (0.54–2.25)	2.78 (2.00–3.87)
Wang et al., 2022 [18]	cardiac surgery	3171/18	1.08 (0.91–1.29)	2.14 (1.41–3.24)
DeCassai et al., 2022 [17]	LCC		1.66 (0.92–2.9)	2.81 (1.34–5.91)
Lewis et al., 2022 [19]	mechanical ventilation in ICU	11997/77	1.32 (1.07–1.63)	2.39 (1.82–3.13)
Kin Sin et al., 2022 [20]	Non-cardiac, non-neurosurgical operations	2676/32	5.39 (1.12–5.89)	5.13 (0.96–27.47)

**Table 2:** Summary of controlled experimental studies on the effects of DEX on myocardial protection. ↑ = increased, ↓ = decreased, → = followed by. Yohimbine is an α2-adrenergic antagonist.

Study	Species/Model	DEX Effects	Mechanisms
Bunte et al., 2020 [41]	rat/isolated heart coronary flow stopped chemically for 33 min → reperfusion 60 min	↓ infarct size by 60% on average	postconditioning
Yin et al., 2020 [42]	rat/embryonic heart-derived myoblast exposed to H2O2	↑ HO-1 and ↓ RIPK1 and RIPK3 expression ↓ necroptosis (reversed by yohimbine) ↓ apoptosis (yohimbine had no effect)	preconditioning reduction of oxidative stress partly α2-adrenergic mechanism
Li et al., 2021 [43]	rat/LAD ligation for 30 min → reperfusion 120 min Functional, biochemical and histological examination	LVEF ↑ by ≈30%, S-LDH, CKMB and TnT ↓, activation of PI3K/Akt pathway, upregulation of Beclin1 phosphorylation	decreased autophagy
Zhong et al., 2020 [44]	(a) rat/LAD ligation for 30 min → reperfusion 120 min (b) rat/myocardial cells histology	↓ pyroptosis ↑ NLRP3, ASC and cleaved-caspase-1	FoxO3a/ARC axis activation
Yang et al., 2021 [45]	rat/ischaemia-reperfusion injury	↓ cytotoxicity in cardiomyocytes ↓ ERS signaling pthw, ↓ GRP78, PERK, CHOP, IRE1 ↑ ATF6 yohimbine inhibition	inhibition of inflammation and apoptosis α2-adrenergic mechanism
Huang et al., 2021 [46]	rat/cardiomyocytes exposed for hypoxia 6 h → reoxygenation 4 h	↑ beating rates and viability of cardiomyocytes ↑ expression of the NLRP3 protein ↓ expression of Bcl2 and BAX ↓ IL-1β, IL-18 and TNF-α and H/R-induced NLRP3 inflammasome activation	inhibition of inflammatory response
Xiong et al., 2021 [47]	rat/LAD ligation for 30 min → reperfusion 120 min; in vivo: cardiac ultrasound for hemodynamics, in vitro: cell histology	↓ degranulation of mast cells and the apoptosis of cardiomyocytes ↓ inhibits the activation of inflammatory related factors HMGB1, TLR4 and NF-κB p65	preconditioning
Sun et al., 2021 [48]	mice/ischemia model	↑ EF and FS 7 d after infarction ↓ expression of ROCK1 and 2 protein ↓ apoptosis	inhibition of the RhoA/ROCK signaling pathway
Xiao et al., 2021 [49]	Human ventricular tissue from tetralogy of Fallot (TOF) patients and CMs derived from human-induced pluripotent stem cells/reperfusion injury and hypoxic injury	↑ AMP-activated protein kinase (AMPK) and phospho AMPK (pAMPK) during the I/R process inhibited by yohimbine	α2-adrenergic mechanism in suppression of autophagy
Song et al., 2021 [50]	rat/LAD ligation for 60 min → reperfusion for 120 min	preconditioning average infarct size ↓30% yohimbine prevented	bradykinin receptor upregulation α2-adrenergic mechanism
Shen et al., 2021 [51]	swine/resuscitation for 5 min after surgically induced cardiac arrest of 8 min	↓ inflammation, oxidative stress, and cell apoptosis and necroptosis in the heart and brain	postconditioning

Abbreviations: apoptosis-associated speck-like protein containing CARD (ASC), activating transcription factor 6 (ATF6), glucose-regulated protein 78 (GRP78), heme oxygenase 1 (HO-1), protein kinase R-like endoplasmic reticulum kinase (PERK), C/EBP homologous protein (CHOP), inositol-requiring protein 1 (IRE1), NOD-, LRR- and pyrin domain-containing protein 3 (NLRP3), receptor interacting protein kinase 1 (RIPK1), receptor interacting protein kinase 3 (RIPK3) silent information regulator factor 2-related enzyme 1 (SIRT1)/mammalian target of rapamycin (mTOR), left ascending coronary artery (LAD), ischemia/reperfusion (I/R), TnT (troponin-T), LDH (lactate dehydrogenase), CKMB (myocardial creatine kinase), PI3K/Akt (phosphoinositide-3-kinase–protein kinase B).

**Table 3:** Hemodynamics and oxygenation measurements before (pre) and after (post) DEX infusion. Median values (and ranges) are given. Pre = baseline, Post = after DEX.

Timing	SAP (mmHg)	DAP (mmHg)	MAP (mmHg)	SpO2 (%)	Hr (b/min)
Pre	125 (109–156)	78.5 (67–106)	94.5 (91–98)	95 (91–98)	69 (52–100)
Post	122 (99–145)	81 (69–95)	93.5 (84–112)	93.5 (91–97)	73 (51–92)

**Table 4:** Electrophysiological properties. Figures represent time in milliseconds (ms). Pre = baseline, Post = after DEX. Statistically significant difference between pre- and post-DEX measures is marked with * (*p* < 0.05).

Variable	Pre (ms)	Post (ms)
P (duration of P-wave)	125 (105–155)	121 (103–155)
PQ (PQ interval)	172 (133–201)	176 (133–205)
QRS (duration of QRS)	94 (83–123)	97 (80–124)
QTc (duration of QT-interval)	416 (382–473)	421 (398–468)
SCL (sinus cycle length)	913 (671–1179)	941 (762–1082)
SNRT (Sinus node recovery time)	1291 (1012–1744)	1340 (1110–1806)
CSNRT (Corrected sinus node recovery time)	377 (250–734)	468 (144–742) *
AVNW (cycle length producing AV nodal Wenckebach antegrade conduction block)	340 (310–430)	365 (320–420)
AVNERP600 (AV nodal refractory time at 600 millisecond atrial pacing)	270 (230–350)	290 (240–370) *
AVNERP400 (AV nodal refractory time at 400 millisecond atrial pacing)	290 (240–360)	285 (270–340)
AERP (atrial effective refractory period)	250 (180–280)	260 (210–280)
AVNWret (cycle length producing AV nodal Wenckebach retrograde conduction block)	455 (310–680)	505 (340–690) *
AVNERPret (retrograde AV nodal refractory period)	305 (220–430)	290 (220–420)
VERP (Ventricular effective refractory period)	240 (220–260)	240 (220–260)

## Data Availability

The data presented in this study are available on request from the corresponding author. The data are not publicly available due to national ethical restrictions.

## Trial registration

EudraCT 2010-020782-24.
